# Metabolic Reprogramming and Immune Metabolism in Sepsis: Targeting the PPAR Pathway for Personalized Therapeutic Approaches

**DOI:** 10.1155/ppar/2166122

**Published:** 2026-05-09

**Authors:** Zheqian Wu, Yisheng Chen, Lili Yin, Liming Zhu, Fei Ding, Lihua Dai

**Affiliations:** ^1^ Department of Emergency, Shidong Hospital of Yangpu District, Shanghai, China; ^2^ Department of Vascular and Interventional Radiology, Ningde Municipal Hospital of Ningde Normal University, Ningde, Fujian, China; ^3^ Fujian Key Laboratory of Toxicant and Drug Toxicology, Medical College, Ningde Normal University, Ningde, Fujian, China, ndnu.edu.cn; ^4^ Fujian Key Laboratory of Medical Bioinformatics, Fujian Medical University, Fuzhou, Fujian, China, fjmu.edu.cn; ^5^ School of Life Sciences, Fudan University, Shanghai, China, fudan.edu.cn; ^6^ Emergency and Intensive Care Unit, Shidong Hospital of Yangpu District, Shanghai, China

**Keywords:** immune regulation, metabolic intervention, metabolic reprogramming, personalized therapy, PPAR pathway, sepsis

## Abstract

Sepsis is a life‐threatening organ dysfunction caused by a dysregulated host response to infection, in which metabolic reprogramming plays a critical role in disease progression and organ failure. Metabolic reprogramming involves alterations in glucose, lipid, and protein metabolism, leading to imbalanced energy production, immune dysregulation, and tissue damage. Immune cells, under septic stress, switch to aerobic glycolysis, enhancing energy production but causing lactate accumulation and mitochondrial dysfunction, which exacerbates inflammation and organ injury. This metabolic shift emphasizes the need for personalized therapeutic strategies that address the metabolic heterogeneity between pathogens and hosts. The peroxisome proliferator‐activated receptor (PPAR) pathway serves as a central regulator of both metabolic and immune responses, offering protective effects through the promotion of fatty acid oxidation and suppression of inflammation. However, the translational application of PPAR‐directed therapies is constrained by limited drug specificity and significant interpatient heterogeneity. Advances in multiomics technologies provide promising opportunities for identifying metabolic biomarkers and tailoring PPAR‐targeted treatments. Future research should focus on integrating metabolic pathways, developing precise diagnostic tools, and refining personalized interventions to improve sepsis management and patient prognosis. Unlike previous reviews that primarily focus on general immunometabolic alterations in sepsis, this review is the first to systematically integrate PPAR isoform‐specific regulatory mechanisms, multiomics‐based patient stratification, and phenotype‐driven therapeutic targeting, thereby offering a novel framework for precision medicine in sepsis management. We critically evaluate the controversies on the efficacy of PPAR agonists, highlight cross talk with HIF‐1*α*/NF‐*κ*B/Nrf2, and propose a phenotype‐based stratification for sepsis therapy, a perspective that has not been explored in the literature.

## 1. Introduction

Sepsis, a severe systemic inflammatory response to infection, remains a leading cause of death in critically ill patients, with over 49 million cases and 11 million deaths annually worldwide [1]. Despite advances in early diagnostic approaches and supportive care technologies, sepsis‐related mortality has shown minimal improvement, primarily owing to the complex and multifactorial characteristics of its underlying pathophysiology [2, 3]. A central hallmark of sepsis is metabolic reprogramming, which orchestrates immune responses, inflammatory signaling, and organ function [4]. This reprogramming reflects adaptive shifts in how cells produce and distribute energy during infection, closely linked to the immune system and inflammatory activity [5]. A more thorough understanding of these metabolic processes is critical for uncovering new therapeutic strategies to combat sepsis [6]. During sepsis, host immune cells undergo a rapid metabolic shift to effectively combat invading pathogens and withstand immunological stress [7]. This transformation involves alterations in energy metabolism, utilization of carbon sources, mitochondrial efficiency, and redox balance regulation [8]. The hallmark of this adaptation is activation of aerobic glycolysis, the so‐called Warburg effect, in which immune cells ramp up the metabolism of glucose to produce ATP and biosynthetic precursors [9]. Although this shift supports enhanced immune responsiveness, it concurrently drives excessive lactate accumulation, disturbs acid–base equilibrium, and contributes to immune dysfunction, ultimately worsening organ injury and heightening the risk of mortality [10]. Consistent findings from integrated multiomics studies emphasize metabolic reprogramming as a key factor in sepsis pathogenesis, reinforcing its potential for guiding the development of diagnostic markers and targeted therapies [11–15].

The metabolic shifts induced during sepsis differ depending on the nature of the invading pathogen, as infections caused by Gram‐positive bacteria, Gram‐negative bacteria, fungi, or viruses each provoke distinct metabolic phenotypes [16]. Additionally, patients with underlying conditions such as diabetes or metabolic diseases experience more severe metabolic disruptions and a faster disease progression [17]. This highlights the need for personalized treatment approaches based on metabolic profiles. Targeting metabolic pathways may complement current anti‐infection therapies and offer new avenues for reducing sepsis‐related mortality [18–20]. Artificial intelligence, combined with big data, can also be used to analyze patients’ metabolic characteristics in depth to accurately predict disease risks, thereby providing stronger support for the development of personalized treatment plans [21].

The systemic inflammatory response in sepsis leads to significant disruptions in glucose, lipid, and protein metabolism, which affect cellular function, tissue repair, and organ maintenance [22]. In glucose metabolism, sepsis activates glycolysis, even under sufficient oxygen conditions, increasing immune cell function but leading to reduced ATP production, lactate buildup, and acidosis [23]. In this process, regulating glucose metabolic homeostasis through short fiber microsphere scaffolds can improve immune cell function, helping restore metabolic balance and alleviate the negative impact of metabolic disorders on immune response and organ function [24]. Lipid metabolism is also disrupted, with enhanced lipolysis and impaired fatty acid oxidation contributing to lipotoxicity and organ damage, especially in the liver, heart, and kidneys [25]. This dysfunction exacerbates inflammation through lipid derived mediators, further amplifying the immune response. Protein metabolism in sepsis is characterized by increased protein degradation and reduced synthesis, leading to muscle atrophy, protein malnutrition, and impaired immune function [26]. With the increasing importance of posttranslational modifications of proteins in metabolic diseases, studies that combine bioinformatics approaches to predict modification sites and functions can explore new targeted therapeutic strategies [27–29].

Metabolic reprogramming within immune cells plays a pivotal role in modulating immune responses during sepsis [30]. These metabolic transitions exert direct control over immune cell fate decisions, activation states, and effector functions [31]. For example, macrophages primarily rely on glycolysis not only for energy production but also to fuel the synthesis of pro‐inflammatory mediators, whereas de novo fatty acid synthesis plays a vital role in supporting cytokine generation [32]. Mitochondrial damage, which is typically caused by inflammation and the oxidative stress of immune cells, can aggravate tissue damage [33]. Moreover, metabolic intermediates such as succinate and lactate serve as bioactive signaling molecules that modulate both pro‐inflammatory responses and immunosuppressive pathways, thereby increasing the likelihood of secondary infections and aggravating disease progression [34]. Within the context of sepsis, immune cells exhibit distinct metabolic preferences that directly influence their functional capacities [35]. Effector T cells predominantly utilize glycolysis to facilitate their swift activation and proliferation, while regulatory T cells rely more heavily on fatty acid oxidation and oxidative phosphorylation to sustain their immunosuppressive activities [36]. These contrasting metabolic pathways play a pivotal role in modulating immune equilibrium and overall response efficiency. When sepsis emerges, it can disrupt these metabolic pathways [37]. These disruptions hamper the appropriate differentiation of immune cells, which can lead to a state of chronic inflammation or immune paralysis. Both consequences can be very detrimental to disease outcome and patient prognosis [38]. This review takes metabolic reprogramming as a central entry point to systematically elucidate the differential roles of peroxisome proliferator‐activated receptor (PPAR) isoforms in immunometabolic regulation during sepsis and their crosstalk with key signaling networks, including hypoxia‐inducible factor 1*α* (HIF‐1*α*), nuclear factor *κ*B (NF‐*κ*B), and nuclear factor erythroid 2–related factor 2 (Nrf2). By integrating multiomics technologies with metabolic phenotype heterogeneity, we propose a PPAR‐centered conceptual framework for phenotype‐based stratification and precision intervention. Through the synthesis of isoform‐specific mechanisms, omics‐driven patient stratification, and phenotype‐oriented therapeutic strategies, this review provides a novel theoretical paradigm for precision metabolic therapy in sepsis [39].

## 2. Changes in Metabolic Pathways in Sepsis

### 2.1. Metabolic State Sensing: Clinical Manifestations of Energy Imbalance

**Figure 1:**
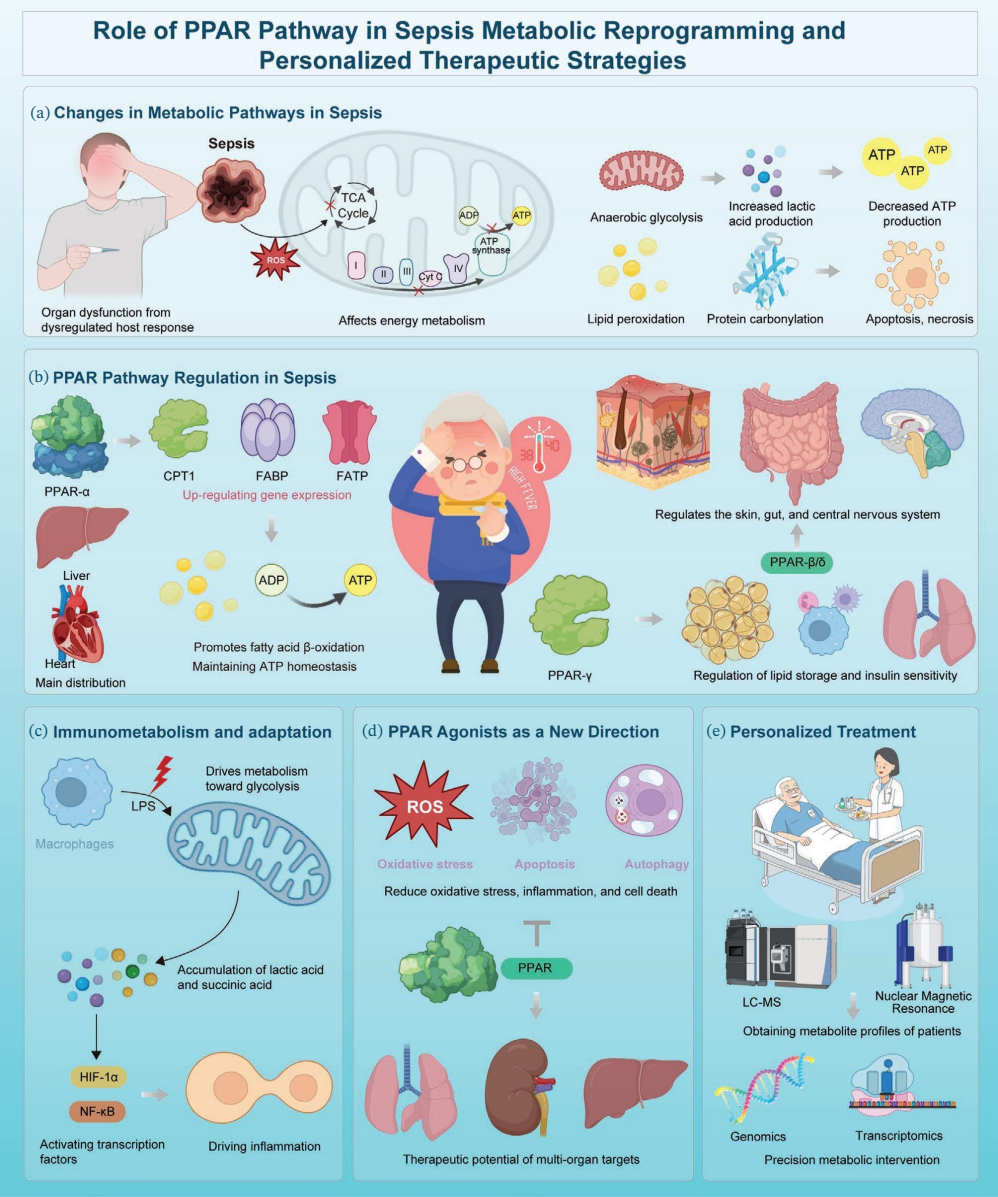
Role of PPAR pathway in sepsis metabolic reprogramming and personalized therapeutic strategies. (a) Sepsis triggers mitochondrial dysfunction, impairing the TCA cycle and ATP production, increasing ROS, and shifting cells toward glycolysis, causing lactic acidosis and cell death. (b) PPARs (*α*, *γ*, *β*/*δ*) regulate energy metabolism. PPAR‐*α* enhances fatty acid oxidation in the liver and heart; PPAR‐*γ* supports lipid storage and insulin sensitivity; PPAR‐*β*/*δ* aids energy balance in various tissues. (c) LPS‐activated macrophages shift to glycolysis, increasing lactate and succinate, activating HIF‐1*α* and NF‐*κ*B, driving inflammation. (d) PPAR agonists (e.g., fenofibrate, pioglitazone) reduce oxidative stress, inflammation, and cell death while promoting mitochondrial function. (e) Multiomics technologies (e.g., LC‐MS, NMR, transcriptomic) enable precise profiling of immune‐metabolic states, supporting personalized sepsis treatment.

Sepsis is characterized by severe metabolic dysregulation, most notably reflected in disrupted energy balance and mitochondrial impairment [40]. The strong inflammatory response, especially the cytokine storm due to mediators like TNF‐*α*, IL‐1*β*, and IL‐6, causes insulin resistance and blocks glucose uptake in cells [41]. This metabolic disruption causes increased blood glucose levels and decreased efficiency of glucose utilization for energy production. Therefore, more and more glucose is converted to energy through anaerobic glycolysis. This process does not produce much energy, and therefore leads to the accumulation of lactic acid. At the same time, mitochondrial dysfunction exacerbates energy deficits by reducing ATP synthesis and elevating the production of reactive oxygen species (ROS), which in turn damage mitochondria and other intracellular structures, ultimately accelerating apoptotic cell death (Figure 1). The onset and progression of multi‐organ dysfunction syndrome can be mainly due to this metabolic collapse. Disruption in lipid metabolism, especially defective fatty acid oxidation, leads to the accumulation of free fatty acids and ketone bodies that could enhance inflammation through Toll‐like receptor activation [41, 42]. The failure of metabolic adaptation in sepsis patients, even with adequate nutrition, suggests that the metabolic mechanisms themselves are compromised, and metabolic phenotyping may help guide personalized treatment strategies. In summary, the breakdown of metabolic regulation is a key driver of sepsis progression and organ failure.

### 2.2. Metabolic Immune Cross‐Regulation

Metabolic reprogramming in sepsis plays a central role in immune cell activation and function [43, 44]. Inflammatory signals induce immune cells, particularly monocytes, macrophages, and neutrophils, to switch from oxidative phosphorylation to glycolysis, supporting their heightened immune activity [10, 45, 46]. Glycolytic intermediates, such as succinate and lactate, regulate pro‐inflammatory pathways, amplifying the immune response [47]. Succinate stabilizes HIF‐1*α* and enhances IL‐1*β* release, whereas citrate promotes the generation of pro‐inflammatory lipid mediators. Notably, PPAR‐*γ* activation has been shown to antagonize HIF‐1*α* signaling by promoting its degradation, thereby attenuating glycolytic flux and inflammatory cytokine production. Conversely, PPAR‐*α* can suppress NF‐*κ*B activation by inhibiting I*κ*B kinase activity and reducing the transcription of pro‐inflammatory genes. ROS production further activates classical inflammatory pathways, including NF‐*κ*B, which in turn can inhibit PPAR transcriptional activity, forming a feedback loop that exacerbates metabolic dysregulation. In lipid metabolism, macrophage polarization is influenced by fatty acid oxidation, with M2 macrophages being anti‐inflammatory and M1 macrophages promoting inflammation [48]. AMP‐activated protein kinase (AMPK) regulates this balance, with reduced AMPK activity linked to immune suppression [49]. Chronic sepsis may lead to a “low metabolism immune suppression” state, characterized by immune cell exhaustion and increased susceptibility to secondary infections. Targeting metabolic‐immune interactions through strategies like glycolysis inhibitors and mitochondrial enhancers offers potential for improving immune function and sepsis outcomes [50].

### 2.3. PPAR Pathway and Metabolic Regulation

PPARs are critical regulators of metabolism and inflammation [51]. The three members PPAR‐*α*, PPAR‐*γ*, and PPAR‐*β*/*δ* have specific yet coordinated roles in sepsis that rely on their ability to regulate metabolic and immune pathways differently (Figure 1) [52–54]. PPAR‐*α* is predominantly expressed in the liver and skeletal muscle, where it enhances fatty acid oxidation and ketogenesis, thereby sustaining energy supply during septic stress [55]. Its downregulation in sepsis contributes to hepatic lipid accumulation, oxidative stress, and impaired neutrophil function [56]. In contrast, PPAR‐*γ* is highly expressed in adipose tissue and macrophages, where it promotes lipid storage, insulin sensitivity, and alternative macrophage polarization toward the M2 phenotype, facilitating inflammation resolution [57–59]. PPAR‐*β*/*δ*, widely expressed in skeletal muscle, skin, and immune cells, enhances oxidative metabolism, mitochondrial biogenesis, and vascular integrity, while suppressing NF‐*κ*B‐driven inflammation. These isoform‐specific actions highlight the potential for targeted PPAR modulation in sepsis [60]. At the same time, by enhancing mitochondrial function and insulin sensitivity, PPAR‐*δ* activation can improve sepsis outcomes. The therapeutic potential of PPAR‐based treatments, in combination with other metabolic interventions, is being explored and may provide a multitarget approach to managing sepsis.

## 3. PPAR Pathway Regulation in Sepsis

Sepsis, characterized by a systemic inflammatory response to infection, triggers a cycle of metabolic dysregulation and immune imbalance [61]. Recent studies highlight the essential role of PPARs in sepsis progression through their regulation of metabolic and inflammatory responses [62]. Despite promising preclinical data, the efficacy of PPAR agonists in sepsis remains contentious [63]. For instance, fenofibrate improves survival in endotoxemia models but shows variable efficacy in polymicrobial sepsis, suggesting model‐dependent and context‐specific responses. Moreover, most agonists lack isoform selectivity, leading to off‐target effects. Essential issues include the lack of adequately powered randomized controlled trials (RCTs) and insufficient consideration of patient metabolic heterogeneity, both of which need to be addressed before broader clinical application. Pharmacological studies have yielded inconsistent results, in part because of differences in dosing schedules, experimental models, isoform‐specific effects, and the lack of isoform‐selective agonists. Similarly, rosiglitazone shows anti‐inflammatory benefits but is associated with fluid retention and cardiovascular risks in susceptible populations. These discrepancies underscore the need for more standardized preclinical models and rigorous pharmacokinetic‐pharmacodynamic assessments to clarify the therapeutic window of PPAR agonists in sepsis. The increasing understanding of the dynamic PPAR signaling network has provided a basis for a metabolic‐immune dual‐target strategy, which may shift therapeutic approaches from traditional organ support to mechanism‐based interventions.

### 3.1. PPAR Isoforms in Sepsis

PPARs are nuclear receptor transcription factors, which contain a conserved structure with four functional domains. They have an N‐terminal ligand‐independent activation region (AF‐1), a DNA‐binding domain (DBD), a hinge region as well as a ligand‐binding domain (LBD) having a ligand‐dependent activation function (AF‐2). The PPAR‐*α*, PPAR‐*β*/*δ*, and PPAR‐*γ* isoforms differ in their tissue distribution, affinity for ligands, and physiological function [64]. In the context of sepsis, PPARs exert protective effects by modulating both metabolic pathways and immune responses (Figure 1). PPAR‐*α* predominantly enhances FAO in hepatocytes and neutrophils, thereby supporting energy supply and reducing lipid toxicity [65]. PPAR‐*γ*, in contrast, induces macrophage polarization toward the M2 phenotype through the upregulation of arginase‐1 and represses glycolytic genes through the inhibition of HIF‐1*α*. PPAR‐*β*/*δ* influences mitochondrial metabolism and thereby contributes to the regulation of immune responses. PPAR‐*β*/*δ* enhances mitochondrial fatty acid oxidation and oxidative phosphorylation in myocytes and endothelial cells, thereby supporting energy homeostasis and reducing endothelial activation. Importantly, PPAR‐*β*/*δ* also modulates T cell metabolism by favoring regulatory T cell differentiation over Th17 responses, illustrating its immunometabolic versatility in sepsis. Collectively, the complementary actions of PPAR isoforms present a synergistic therapeutic strategy for managing sepsis. Therefore, the development of selective agonists targeting these specific isoform actions represents a promising therapeutic approach [65, 66].

### 3.2. PPARs and Clinical Features of Sepsis

Sepsis is often accompanied by marked metabolic derangements such as insulin resistance and hypertriglyceridemia, conditions that are strongly associated with impaired PPAR signaling [67]. In critically ill individuals, the expression of PPAR‐*α* is significantly diminished, leading to reduced fatty acid oxidation and consequent energy shortages [68]. Likewise, decreased PPAR‐*γ* activity enhances lipolysis, resulting in abnormal lipid deposition within nonadipose tissues [69]. These shifts in PPAR expression not only reflect underlying metabolic imbalance but also hold promise as stratification biomarkers for sepsis; for example, circulating sCD163 levels and markers of PPAR‐*α* activity have demonstrated correlations with patient prognosis [70, 71]. Emerging clinical trial data support the therapeutic benefit of PPAR‐*α* and PPAR‐*γ* agonists in sepsis, highlighting the promise of precision medicine approaches that target the PPAR pathway to enhance treatment efficacy.

The use of PPAR modulators like fenofibrate in clinical settings has demonstrated potential in improving patient outcomes by optimizing metabolic regulation and mitigating organ dysfunction [72]. Furthermore, the development of tissue‐specific PPAR agonists is aimed at reducing systemic adverse effects, enhancing therapeutic precision and safety [73]. Advances in technologies like single‐cell sequencing are revealing significant heterogeneity in PPAR expression across immune cells in sepsis, supporting the development of tailored intervention strategies [74–76]. Future research should integrate metabolomics, epigenetics, and AI to refine the dynamic PPAR signaling map, paving the way for precision treatments in sepsis [77].

## 4. Interaction of Metabolic Pathways and Metabolic Adaptation

### 4.1. Synergistic Mechanism Between Metabolic Adaptation and Immune Response

**Figure 2:**
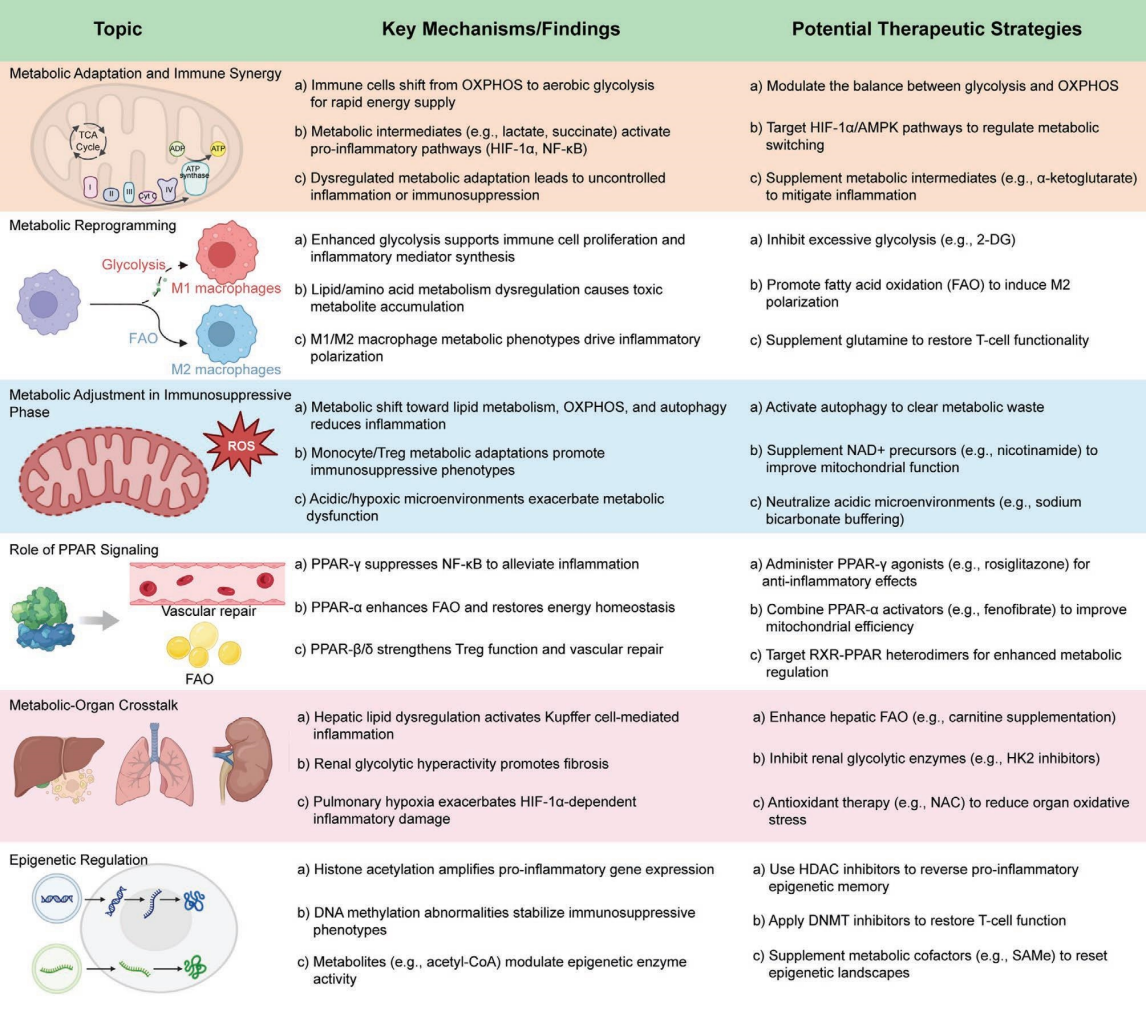
Interactions of metabolic pathways and metabolic adaptation in sepsis. This figure shows the metabolic changes in sepsis and their impact on immune responses. In sepsis, immune cells shift to glycolysis, promoting inflammation. Targeting pathways like HIF‐1*α* and AMPK may help restore balance. Increased glycolysis supports immune activation but also worsens inflammation, making metabolic modulation important for controlling immune responses. In later stages, lipid metabolism and oxidative phosphorylation dominate, leading to immunosuppression. Restoring mitochondrial function could improve recovery. PPAR signaling helps regulate inflammation and metabolism, and activating PPARs may reduce tissue damage. Metabolic dysfunction in organs like the liver, kidneys, and lungs contributes to systemic inflammation, with targeted therapies showing promise. Epigenetic changes also influence immune responses, suggesting potential for epigenetic‐based treatments.

Sepsis induces widespread reorganization of immune, inflammatory, and metabolic networks. Immunometabolism research reveals that metabolic pathways not only support energy needs but also regulate immune responses [78]. During the acute phase, immune cells rapidly reprogram metabolism, shifting from oxidative phosphorylation to glycolysis to meet energy and biosynthetic demands [79]. This adaptation is especially evident in macrophages and T cells, where glycolytic intermediates such as lactate and succinate activate transcription factors like HIF‐1*α*, enhancing inflammatory responses (Figure 2) [80]. PPAR‐*γ* directly competes with HIF‐1*α* for transcriptional coactivators, thereby repressing glycolytic genes such as LDHA and PDK1. Meanwhile, PPAR‐*α* antagonizes NF‐*κ*B by enhancing I*κ*B*α* synthesis and inhibiting IKK phosphorylation. PPAR‐*β*/*δ* synergizes with Nrf2 to activate ARE‐driven antioxidant genes, mitigating sepsis‐associated oxidative stress. This cross‐talk positions PPARs as nodal regulators of immunometabolic balance, which is crucial for mitigating sepsis‐associated oxidative damage. As sepsis progresses, metabolic states shift toward pathways promoting resolution and tissue repair, including lipid metabolism and autophagy. This metabolic flexibility is essential for immune cell plasticity and survival. Key regulators like HIF‐1*α*, mTOR, and AMPK control this dynamic by sensing environmental stress and adjusting gene expression. Metabolic adaptation thus represents a central node linking immune function and inflammatory progression, and its dysregulation is pivotal in the transition to multi‐organ dysfunction [81].

### 4.2. Metabolic Reprogramming and Immune System Function

Metabolic reprogramming enables immune cells to meet functional demands under septic conditions [82]. Initially, macrophages, dendritic cells, and T cells rely on enhanced glycolysis and lipid synthesis to support proliferation and cytokine production [83]. However, prolonged activation may lead to oxidative stress and tissue injury. M1 macrophages dominate early responses with glycolytic metabolism, whereas M2 macrophages favor fatty acid oxidation to support repair (Figure 2) [84]. In sepsis, this balance is disrupted, leading to immune dysfunction. Similarly, activated T cells require glycolysis, but persistent inflammation leads to metabolic exhaustion, impaired cytokine synthesis, and Treg expansion, promoting immunosuppression. Metabolic disturbances also affect B cells and organ function, creating a vicious cycle of inflammation and dysfunction [85, 86]. Stable epigenetic changes lock immune cells in dysfunctional states, underscoring the need for therapies that go beyond anti‐inflammatory strategies to include metabolic modulation.

### 4.3. PPARs and Immune Metabolic Adaptation

PPARs are key nuclear receptors that regulate lipid metabolism and immune responses [87]. Among them, PPAR‐*γ* modulates inflammation by suppressing NF‐*κ*B and promoting anti‐inflammatory macrophage polarization. PPAR‐*α* enhances mitochondrial function and energy efficiency, supporting immune resilience during sepsis [88]. PPAR‐*β*/*δ* contributes to tissue repair and vascular protection. Activation of these receptors modulates gene expression involved in metabolism, redox balance, and immune regulation [89]. However, sepsis‐associated stress impairs PPAR signaling, exacerbating metabolic dysfunction. Despite promising preclinical evidence, the therapeutic efficacy of PPAR agonists in sepsis remains unvalidated by large‐scale RCTs. A major limitation is the lack of selective agonists that can target specific PPAR isoforms without off‐target effects, underscoring the need for more precise pharmacologic tools. Thus, targeting PPARs offers a potential therapeutic strategy for modulating immune metabolism in sepsis.

## 5. Prospects and Challenges of Metabolic Therapy in Sepsis

### 5.1. Limitations of Current Treatment Strategies and Metabolic Interventions

Sepsis, a complex syndrome initiated by infection, leads to systemic inflammation and can progress to multiple organ dysfunction [90]. The underlying pathophysiology of sepsis involves immune dysregulation, metabolic abnormalities, mitochondrial dysfunction, and microcirculatory disturbances [91]. Although current treatments, including broad‐spectrum antibiotics, fluid resuscitation, hemodynamic support, and immune modulation, have reduced early mortality, their efficacy diminishes when sepsis progresses to immune suppression and severe metabolic dysfunction. Research demonstrates that sepsis involves a dynamic disruption of immune‐metabolic balance, underscoring the shortcomings of conventional anti‐inflammatory treatments, which do not effectively target cellular metabolic disturbances such as mitochondrial dysfunction and reduced ATP synthesis [92]. Emerging metabolic therapies seek to restore energy homeostasis, enhance the coupling between immune function and metabolism, and correct cellular metabolic defects. Nonetheless, significant challenges remain, especially concerning optimal treatment timing, precise therapeutic targets, and personalized intervention strategies.

A significant challenge lies in the ineffectiveness of traditional anti‐inflammatory therapies once patients enter an immune‐suppressed state marked by T cell exhaustion and diminished immune function [93]. These therapeutic approaches fail to address the fundamental metabolic disturbances that compromise immune cell function, thereby restricting their overall clinical efficacy. Moreover, many existing metabolic interventions, such as glucose administration or amino acid supplementation, are largely empirical and overlook the metabolic heterogeneity present among sepsis patients [94]. Without precision‐guided approaches, these interventions risk aggravating metabolic imbalances. The lack of dependable monitoring tools and biomarkers to guide metabolic interventions further restricts their application in clinical settings. Although critical metabolic signaling pathways such as mTOR, AMPK, and HIF‐1*α* have been recognized for their roles in sepsis development, their incorporation into routine clinical practice remains limited [95]. These pathways are vital for energy sensing, regulation of oxidative stress, and immune cell function, yet comprehensive clinical validation is still required. Moreover, metabolic modulation therapies may produce effects that vary depending on the disease stage and carry the risk of adverse reactions, which complicates their clinical translation [96].

### 5.2. PPAR Agonists as a New Direction for Sepsis Treatment

Recent investigations have highlighted the promising role of PPAR agonists in sepsis management, largely due to their ability to modulate energy metabolism, inflammation, and immune responses [88]. Members of the PPAR family, especially PPAR‐*α* and PPAR‐*γ*, have been extensively studied for their therapeutic relevance in metabolic diseases, and their importance in sepsis is gaining increasing recognition [97]. These nuclear receptors regulate lipid metabolism, glucose utilization, and inflammatory signaling pathways. A systematic analysis of preclinical studies reveals that PPAR‐*α* agonists consistently improve survival in murine endotoxemia but show limited efficacy in polymicrobial models. Conversely, PPAR‐*γ* agonists like rosiglitazone exacerbate fluid retention in susceptible populations, highlighting clinical trade‐offs. Table 1 summarizes agonist mechanisms and translational status, emphasizing the urgent need for isoform‐selective agents and biomarker‐guided trials. A critical evaluation of available clinical trials indicates that although some studies report improved metabolic parameters and reduced cytokine levels with PPAR agonist use, none have yet demonstrated a conclusive mortality benefit in large RCTs [98]. Moreover, issues such as patient heterogeneity, timing of intervention, and agonist selectivity complicate the interpretation of these findings. Future RCTs should prioritize patient stratification based on metabolic phenotypes and PPAR expression profiles to better evaluate therapeutic efficacy [99]. PPAR‐*α* agonists may improve septic outcomes by enhancing hepatic and myocardial fatty acid oxidation, reducing lipotoxicity, and restoring NAD^+^ to support mitochondrial function, whereas PPAR‐*γ* agonists dampen inflammation by promoting M2 macrophage polarization and inhibiting pyroptosis via suppression of HIF‐1*α* and NF‐*κ*B signaling, suggesting that dynamic monitoring (including imaging‐based readouts where feasible) could help track macrophage reprogramming and optimize the therapeutic window [100]. They also synergize with Nrf2 to bolster antioxidant defenses. Similarly, PPAR‐*α* agonists mitigate inflammation via NF‐*κ*B inhibition and enhance mitochondrial function, whereas PPAR‐*β*/*δ* agonists support vascular integrity and redox homeostasis. However, their clinical use is limited by side effects such as fluid retention and weight gain, underscoring the need for isoform‐selective or tissue‐targeted agents. PPAR‐*β*/*δ* agonists enhance skeletal muscle oxidative metabolism and vascular stability, which may prevent sepsis‐induced cachexia and endothelial dysfunction. The concept of combining isoform‐specific PPAR agonists represents a promising multitarget approach, yet systematic pharmacological evaluations are scarce. Preclinical studies combining PPAR‐*α* and PPAR‐*γ* agonists show enhanced anti‐inflammatory and metabolic effects compared with monotherapy, but also highlight the risk of compounded side effects, such as hepatotoxicity or exacerbated immunosuppression in chronic sepsis phases. A systematic review of combination regimens underscores the necessity of phase‐specific dosing and biomarker‐guided administration. Future studies should integrate pharmacogenomics and real‐time metabolic monitoring to optimize combination strategies and minimize adverse outcomes (Figure 1) [101]. Such mechanisms are critically relevant in sepsis, a disorder marked by severe inflammation and metabolic dysregulation. However, obstacles remain, such as potential side effects and the need for the development of tissue‐specific agonists. Ongoing clinical studies are necessary to refine and optimize the use of PPAR agonists in treating sepsis. To enhance translational relevance, we provide a summary of selected PPAR agonists, their mechanisms, and current preclinical/clinical status in sepsis (Table 1). This table highlights the potential and challenges of targeting PPAR pathways for personalized therapy. As summarized in Table 1, current PPAR agonists exhibit promising preclinical efficacy but face significant translational barriers, including isoform nonselectivity and phenotypic heterogeneity. Future trials must incorporate metabolic biomarkers to enable patient enrichment.

**Table 1:** Summary of PPAR agonists in sepsis research.

PPAR agonist	Target PPAR isoform	Mechanism of action in sepsis	Preclinical evidence	Clinical status
**Fenofibrate**	PPAR‐*α*	Enhances fatty acid oxidation in liver and heart; reduces inflammation via NF‐*κ*B and NLRP3 inflammasome inhibition	Improves survival in rodent endotoxemia models; efficacy inconsistent in polymicrobial sepsis models	Limited data; small‐scale studies show improved metabolic parameters, but no mortality benefit in large RCTs
**Rosiglitazone**	PPAR‐*γ*	Promotes M2 macrophage polarization; suppresses HIF‐1*α* and NF‐*κ*B pathways; inhibits pyroptosis	Anti‐inflammatory benefits in septic models	Associated with fluid retention and cardiovascular risks; no conclusive mortality benefit in sepsis trials
**Pioglitazone**	PPAR‐*γ*	Similar to rosiglitazone; enhances M2 polarization and mitigates organ injury	Reduces organ injury in preclinical studies	Sparse clinical data; primarily from retrospective or small prospective studies
**GW501516**	PPAR‐*β*/*δ*	Enhances mitochondrial biogenesis and oxidative metabolism; modulates T‐cell differentiation toward Treg cells	Preclinical evidence of improved metabolic resilience and vascular integrity	Not evaluated in clinical trials for sepsis

*Note:* A systematic evaluation of PPAR agonists reveals isoform‐specific efficacy and translational challenges. Although fenofibrate shows promise in endotoxemia, its utility in clinical sepsis is unproven. Rosiglitazone′s anti‐inflammatory effects are offset by cardiovascular risks, underscoring the need for selective agonists.

### 5.3. Personalized Treatment and Precision Metabolic Interventions

The inherent complexity and heterogeneity of sepsis underscore the urgent need for personalized therapeutic approaches that account for patient‐specific factors such as genetic variations in PPAR signaling, metabolic phenotypes, sex differences, and comorbidities [102]. Given the wide interpatient variability in metabolic responses, uniform treatment often yields inconsistent efficacy. We propose a phenotype‐based stratification framework integrating PPAR genotype, metabolic profiling (glycolytic flux vs. mitochondrial dysfunction), and comorbidities. Patients with high glycolysis and low PPAR‐*α* expression may benefit from PPAR‐*α* agonists, whereas those with mitochondrial dysfunction could respond better to PPAR‐*β*/*δ* activation. Real‐time metabolomics combined with AI decision support could further optimize agonist selection, enabling personalized metabolic interventions aligned with individual metabolic‐immune profiles [103–106]. Multiomics and AI platforms hold promise for advancing personalized sepsis care by integrating genomic, metabolomic, and clinical data to identify patient subgroups with distinct PPAR pathway activities and metabolic phenotypes. However, significant hurdles persist, including data integration across omics layers, algorithmic transparency, and validation in diverse cohorts stratified by genetic background, sex, and comorbidities [107]. To make AI‐enabled stratification clinically reliable, studies should jointly implement standardized phenotype acquisition workflows and multiomics quality‐control strategies, thereby improving reproducibility and transportability across centers [108, 109]. Ultimately, customized metabolic treatments have the potential to revolutionize sepsis care by integrating PPAR genotyping, metabolic phenotyping, and comorbidity profiling into therapeutic algorithms. Future clinical trials should prioritize patient stratification based on PPAR polymorphism status, baseline metabolic profiles, and sex‐specific responses to PPAR agonists to validate precision intervention strategies [110].

## 6. Future Research Directions in Sepsis Metabolic Reprogramming

Sepsis remains a leading cause of death among critically ill patients worldwide [111]. Central to its pathology is metabolic reprogramming, which has attracted increasing attention because of its significant influence on immune responses, cellular functions, and systemic energy metabolism [4]. This complex process involves multifaceted changes at molecular, cellular, and organ levels, each of which is instrumental in advancing sepsis progression.[112]. Future research directions include integrating multidimensional metabolic mechanisms, developing metabolic biomarkers, and translating the PPAR pathway into clinical applications [113].

### 6.1. Integration of Multidimensional Metabolic Mechanisms

Recent advancements in molecular biology have significantly enhanced our understanding of sepsis metabolism. Although traditional research focuses on individual metabolic pathways, sepsis involves alterations across multiple organs and metabolic processes [114]. A future research priority is the integration of multiomics approaches, combining genomics, metabolomics, and epigenetics; it not only provides a more comprehensive perspective on metabolic reprogramming in sepsis, but also helps identify unique metabolic phenotypes in different patient populations, laying the foundation for the development of precision treatment strategies [115–117]. Metabolomics reveals changes in the metabolic profiles of patients, whereas genomics and epigenetics help identify gene variants and regulatory mechanisms that influence metabolic pathways [118]. By combining these disciplines, researchers can gain deeper insights into how metabolic reprogramming occurs at the molecular and cellular levels. Such integrated approaches will not only provide new therapeutic targets but also aid in the development of personalized treatment strategies by translating laboratory findings into clinical practice.

### 6.2. Development of Metabolic Biomarkers and Diagnostic Tools

Early diagnosis is critical for the successful treatment of sepsis, but current diagnostic tools are often inaccurate [119]. Research on biomarkers is key to the early diagnosis and precision treatment of diseases; metabolic biomarkers, which reflect specific metabolic alterations associated with sepsis, offer a promising approach for early detection [120, 121]. Through the integration of multiomics data and bioinformatics methodologies, researchers can pinpoint early biomarkers of sepsis and delineate immune responses within various microenvironments, thereby establishing a comprehensive basis for accurate diagnosis [122–125]. Individuals affected by sepsis exhibit unique metabolic signatures, marked by changes in lipid, amino acid, and glucose metabolism [126]. For example, disruptions in fatty acid metabolism or changes in amino acids such as glutamate and proline are associated with disease severity. The development of biomarkers that accurately reflect these metabolic shifts holds promise for improving early detection of sepsis [127]. Nevertheless, this requires establishing strong and reliable associations between metabolic signatures and clinical conditions to ensure high sensitivity and specificity. Furthermore, advancements in diagnostic technologies, including portable metabolic analyzers and AI‐based predictive models, will play a vital role in facilitating early screening and monitoring of therapeutic responses in sepsis [128].

### 6.3. Clinical Translation of the PPAR Pathway

The PPAR family plays a crucial role in modulating metabolic and immune responses. The three isoforms of PPAR, namely PPAR‐*α*, PPAR‐*β*/*δ*, and PPAR‐*γ*, control the metabolic processes in different tissues related to lipid metabolism, energy homeostasis, and immune regulation [129]. Evidence indicates that activating these receptors can mitigate inflammation, normalize lipid metabolism, and reduce sepsis‐associated tissue injury through intricate cross talk with HIF‐1*α*, NF‐*κ*B, and Nrf2 pathways. For instance, PPAR‐*γ*/Nrf2 coactivation enhances cytoprotective responses, whereas PPAR‐*α*/NF‐*κ*B antagonism alleviates hyperinflammation. Understanding these interactions is crucial for designing combination therapies that target multiple nodes within the immunometabolic network. Although robust preclinical evidence supports PPAR activation as a therapeutic strategy, clinical translation remains challenging. These challenges stem from the context‐dependent dual roles of PPARs in early hyperinflammation versus late‐stage immunosuppression, the limited availability of isoform‐selective agonists, and marked patient heterogeneity. Such heterogeneity arises from genetic variability, metabolic phenotypes, sex differences, and comorbidities such as diabetes or obesity, all of which influence PPAR signaling and therapeutic responsiveness. Consistent with this complexity, a systematic analysis of clinical trial registries indicates that most PPAR‐targeted sepsis studies are small phase I/II trials, often lacking subsequent validation, with outcomes frequently confounded by comorbid conditions and concomitant medications. To overcome these barriers, future adaptive clinical trials should integrate metabolic phenotyping and PPAR pathway biomarkers to enable patient enrichment and stratified analyses [130]. Clinical application is further constrained by adverse effects associated with PPAR agonists, including value="fluid retention" and weight gain [131]. Within this framework, computer‐aided drug design (CADD) emerges as a valuable tool for advancing research by enabling the simulation and screening of potential PPAR modulators, thus providing theoretical and practical support for novel drug development [132]. Further studies should develop selective PPAR modulators. Likewise, further studies must clarify how genotype‐based modifiers of PPARs, baseline metabolic state, accompanying diseases, and other patient‐specific factors shape the contextual duality of PPAR activation along the sepsis timeline. This strategy will allow for PPAR‐targeted therapy tailored to each patient at various stages.

In summary, metabolic reprogramming in sepsis is a complex biological phenomenon encompassing multiple molecular pathways and organ‐level interactions. Future research will necessitate integrated multiomics approaches to elucidate the underlying mechanisms. The discovery of metabolic biomarkers alongside the advancement of sophisticated diagnostic technologies promises to improve early detection and therapeutic monitoring. Additionally, translating PPAR pathway research into clinical practice offers substantial potential, though further studies are necessary to address existing challenges and fully harness its therapeutic benefits in sepsis management.

## Author Contributions

Zheqian Wu and Yisheng Chen contributed equally to this work. Zheqian Wu, Yisheng Chen, and Lihua Dai conceived the study and outlined the review framework. Lili Yin and Fei Ding performed the literature screening and evidence synthesis. Liming Zhu and Lihua Dai contributed to critical revision for important intellectual content.

## Funding

This study was supported by the Ningde City 2024 Talent Introduction Research Start‐up Project (2024Y08), Fujian Province Young Scientific and Technological Talent Development Program (Min Cai Zhi [2025] 0724, 25110100011), Peak Discipline of Yangpu District’s Medical ’Summit‘ Discipline Cluster (25YPGL104), and the Yangpu District Clinical Research Special Project by the Science and Technology and Economy Commission and the Health Commission, titled “Construction and Clinical Translation of an Intelligent Emergency Department Patient Flow Prediction Model Based on Multi‐Modal Environmental Data” (YPM202507).

## Disclosure

All authors reviewed and approved the final manuscript and agree to be accountable for the content and conclusions of the article, ensuring that any questions related to the accuracy or integrity of any part of the work are appropriately investigated and resolved.

## Conflicts of Interest

The authors declare no conflicts of interest.

## Data Availability

Data sharing is not applicable to this article as no datasets were generated or analyzed during the current study.
